# Health system organisation and patient pathways: breast care patients’ trajectories and medical doctors’ practice in Mali

**DOI:** 10.1186/s12889-019-6532-8

**Published:** 2019-02-18

**Authors:** Kirstin Grosse Frie, Bakarou Kamaté, Cheick Bougadari Traoré, Bourama Coulibaly, Brahima Mallé, Eva Johanna Kantelhardt

**Affiliations:** 1 Institute for Medical Epidemiology, Biostatistics and Informatics, Martin-Luther-University Halle-Wittenberg, Magdeburgerstraße 8, 06112 Halle (Saale), Germany; 2 Institute of Pathology, University Hospital Point G, Bamako, Mali; 3 Department of Gynecology, University Hospital Halle (Saale), Halle, Germany

**Keywords:** Breast cancer early detection, Patient pathways, Healthcare-seeking behaviour, Health system, Sub-Saharan Africa

## Abstract

**Background:**

Information on pathways of women seeking diagnostic services due to breast- related symptoms can help highlight challenges related to the healthcare system in improving early diagnosis of breast cancer.

**Methods:**

We retrospectively analysed the entire patient pathway, from first symptom recognition via initial healthcare visit up to final diagnosis at the pathology service in Mali. Data from questionnaire-based structured patient interviews (*n* = 124) were used to calculate time to first healthcare visit (median 91 days) and consecutive time to diagnosis (median 21 days) and to extract information on type of initially visited healthcare facility (community healthcare centre, referral hospital, tertiary hospital, private clinic). Median time to first healthcare visit and time to diagnosis and type of initially-visited healthcare facility were cross-tabulated with patient characteristics. An additional survey among (*n* = 30) medical doctors in the community healthcare centres and referral hospitals in Bamako was conducted to understand current knowledge and referral practice with respect to female patients with breast-related symptoms.

**Results:**

Patients who initially visited private clinics had the shortest time to first healthcare visit (median 44 days), but the longest time to diagnosis (median 170 days). Patients visiting community healthcare centres and referral hospitals took longest for a first healthcare visit (median 153 and 206 days, respectively), but the time to diagnosis was shorter (median 95 and 7 days, respectively). The majority of patients (45%) initially visited a tertiary hospital; these patients had shortest total time to diagnosis (median 56 days health seeking and 8 days diagnostic time), but did not follow the recommended pathway for patients in the pyramidal healthcare system in Mali. The doctors’ survey showed lower breast cancer knowledge in the community healthcare centres than in the referral hospitals. However, most doctors felt able to recognise suspected cases of cancer and referred patients directly to a hospital.

**Conclusions:**

The role of different healthcare facilities in ensuring triage of patients with breast-related symptoms needs to be defined before any early detection initiatives are implemented. Especially at the entry level of the healthcare system, the access and quality of health services need to be strengthened.

## Background

Non-communicable diseases are increasing in sub-Saharan Africa, and the risk of premature death due to heart disease, stroke, cancer, diabetes and chronic lung disease is generally higher than in high-income countries [1]. While primary prevention efforts are necessary to tackle the increasing economic, social and individual burden of non-communicable diseases in sub-Saharan Africa, existing health services need to be strengthened to handle the current demand for diagnosis, treatment and care [2]. Breast cancer is the most prevalent cancer in sub-Saharan Africa, with a high mortality rate [3] and a steady increase in incidence, due to changes in demographics and lifestyles [4]. Since the majority of breast cancer cases are diagnosed at a late stage [5] when treatment is less effective, early detection approaches have gained prominence in the international debate on how to improve survival from the disease in sub-Saharan Africa [6].

Some studies of breast cancer patients in different sub-Saharan African countries [7–10] have analysed factors that lead to late first healthcare visiting and diagnosis – which, in turn, sometimes accounts for detection at a late stage. These investigations consistently have found that a lower level of breast cancer knowledge leads to prolonged time to the first healthcare visit. They commonly recommend awareness and education campaigns to improve symptom recognition and prompt healthcare seeking. However, successful implementations of such programmes would challenge the current healthcare systems in sub-Saharan Africa in terms of providing: (a) adequate triage for all women requiring diagnostic service for breast-related symptoms, and (b) timely diagnosis and treatment, if necessary. In our recent population-based study from Burkina Faso, a neighbouring country of Mali, we estimated that there were currently 184,562 women in the country needing diagnostic service due to breast-related symptoms, but that only 30% of women who reported such symptoms once in their life had sought medical advice.

A recent qualitative study from Mali [11] further revealed that patients who seek healthcare due to breast-related symptoms face several barriers on the individual and health system levels, often leading to a discontinuation of care. An earlier mixed-method study from Ethiopia [12] also showed that breast cancer patients had problems navigating through the complex healthcare system, and patients who initially visited a traditional healer or primary healthcare centre had more consultations before arrival at a tertiary healthcare centre than those who initially visited regional, private or tertiary hospitals [12]. The initial visit to a primary healthcare provider, general surgeon or gynaecologist also increased the risk of delaying diagnosis in a study from Egypt [13]. These investigations assume that, also on the healthcare provider level, certain factors (e.g. low level of breast cancer knowledge among the healthcare providers, wrong diagnoses and no or delayed referral) lead to delays in breast cancer presentation, especially at the level, where women are recommended to enter the system. There is little information on how far patients` socio-demographic characteristics determine the choice of the first healthcare provider among women with breast-related symptoms. In other health contexts in Africa, it was for example described that patients with lower socio-economic status (no education compared to those with at least primary education and those from bigger households with 6 to 10 members, compared to smaller households) were more likely to visit governmental then private healthcare providers [14].

This study retrospectively analysed the pathways of women receiving diagnostic services due to breast-related symptoms in a tertiary hospital in Bamako, Mali, with a focus on the type of healthcare facility patients first visited. Information from patients and medical doctors was used to identify challenges for breast cancer early-detection programmes aimed at improving delivery of timely healthcare visits and diagnosis of patients with breast-related symptoms.

## Methods

### Study setting

The study took place in Bamako, the capital of Mali in Western Africa, and was part of a larger project on breast cancer diagnosis and treatment in Mali [11, 15]. Bamako has a higher density of public and private healthcare facilities at the community level and special services for diagnosis and treatment of breast cancer, compared to the rest of the country. Pathology service is available in Bamako at the University Hospital Point G only. Mali has a pyramidal healthcare system: patients are advised to enter the healthcare system at the community level, from where they are further referred to specialised services (e.g. gynaecology or surgery) at referral and tertiary hospitals. Community healthcare centres are staffed with general medical doctors but, in remote areas, this often is not the case.

### Data collection

#### Patient survey

All women who received diagnostic service due to breast-related symptoms at the Department of Pathology, University Hospital Point G in Bamako, Mali between 1 January and 30 April 2016 were the source population. Patients who consented to participate in the study were interviewed directly by 2 female medical students. The interview took place either on the day the woman received diagnostic service or when she received the results. The students were trained in interview techniques in preparation for the study. All participants provided written or oral informed consent. Ethical clearance was obtained from the local ethics committee of the Medical Faculty in Bamako, Mali.

A standardised questionnaire [15] was used to structure the interview. The instrument was designed to reconstruct the patient pathway from first symptom recognition to diagnosis at the pathology service. It was developed based on instruments used for similar studies in Morocco [16] and Mexico [17], and was adapted for the Malian healthcare setting and in relation to the Model of Pathways to Treatment [18]. Dates of symptom recognition and first healthcare visit were collected with the help of a calendar technique [19], which helps patients recall dates more precisely. The date and type of the first healthcare visit due to the breast-related symptoms were confirmed by medical records, when available. The questionnaire also included items to describe the participant’s health-seeking behaviour and referral pathways. The collected data were linked to socio-demographic and clinical data routinely collected at the pathology service. The questionnaire was written in French but, as most interviews were conducted in Bambara (the local language), it was also orally translated in Bambara together with the 2 interviewers to ensure a common understanding and translation. The questionnaire was then pretested with 4 patients in Bambara in personal interviews. Minor changes helped to improve comprehension of the questions and fluency of the interviews. Each personal interview lasted about 20 min and took place in a quiet room at the pathology service.

#### Healthcare provider survey

Bamako is divided into 6 communities. Each geographical section has from 6 to 12 community healthcare centres (*n* = 56), staffed with a minimum of 1 medical doctor. In each community, there is also 1 referral hospital with specialised services (*n* = 6) [20]. With the consent of each community, 2 medical students who were already involved in the patient interviews, visited a randomly selected sample (*n* = 24) of community healthcare centres and asked the medical doctors on duty to participate in the study. All 6 referral hospitals were visited, and at least 1 medical doctor from the Gynaecology Department of each was invited to be interviewed. If the interview was not possible that day, a new appointment was made that was convenient for the physician. The questionnaire used was developed together with the Malian clinical project cooperation partners. Personal information (age, gender, specialisation, years of work experience, internships in the field of oncology), knowledge of breast cancer (subjectively-rated knowledge) and practice (practice of clinical breast examination, recommendations for suspected cases, and referral praxis) were collected with closed-ended questions. Opinions on how to improve early breast cancer diagnosis and treatment in Mali were collected with one open-ended question.

### Data analysis

#### Patients

Of the 134 patients who had an initial diagnostic workup due to breast-related symptoms at the Pathology Department within the first 4 months of 2016, 4 had no available medical records, 4 did not attend an interview and 2 refused to participate. The final sample included 124 women. Since only women with pathological diagnostic needs were included in our study, and since we assumed that, at the time of symptom recognition and first healthcare visit, no knowledge about the final pathological diagnosis was available, we did not differentiate between cancer or benign pathologic findings in our analysis.

*Health Seeking Interval* (HSI; i.e. time from date of first symptom recognition to date of first healthcare visit) and *Diagnostic Interval* (DI; i.e. time from first healthcare visit to date of receiving results at the Pathology Department) were calculated in days for all participants. Median interval times and initially visited healthcare facility (community health care centre, referral hospital, tertiary hospital or private clinic) were cross-tabulated with patient characteristics (age, marital status, occupation status, residence, health insurance status, knowledge of breast self-examination), health-seeking characteristics (interpretation of the first symptom, visit of a traditional healer, reason to seek health care) and pathologic results (diagnosis, tumour size, lymph node involvement). To further explore the role of the initially-visited healthcare facility, the following information was recorded for patients visiting a community healthcare centre, referral hospital, tertiary hospital or private clinic: median HIS and DI, recommendations received (referral, diagnostic services as mammography, ultrasound and/or fine needle aspiration or only medical treatment), and the mean number of consultations before arriving at the pathology service.

#### Healthcare providers

All invited medical doctors from 24 community healthcare centres, as well as 12 from the 6 referral hospitals, participated in the interviews. Descriptive analyses, stratified by type of medical facility at which the doctors practised, were conducted. The answers to the open-ended question on how to improve timeliness of diagnosis and treatment in Mali were sorted into three broad categories: education, early detection and health services. Frequencies of the respective subcategories were analysed.

## Results

Of 124 women seeking diagnostic services at pathology departments due to any breast-related symptoms in Bamako, Mali, 64 were finally diagnosed with breast cancer, while the rest of the patients were diagnosed with benign lesion, inflammation, other or cancer in situ. Further analysis showed that there were no major differences between cancer and non-cancer patients. Almost all patients (93.5%) reported a breast lump as the first symptom, two-thirds of these women additionally reported pain.

There were large differences in median HSI and DI for certain demographic, healthcare seeking-related, and diagnostic characteristics (Table 1). Furthermore, the patients´ profiles differed by the healthcare facility first visited. Community healthcare centres were mainly visited by married housewives above 50 years, patients without health insurance; or those who initially visited traditional healers after first recognising symptoms and who came because of an aggravation of symptoms. Characteristics of patients in the referral hospitals slightly differed from those in the community healthcare centres. In the referral hospitals the proportion of women with health insurance was higher and less patients sought help from a traditional healer prior to presenting at the hospital. Patients have also been more likely to have received a recommendation from someone to seek healthcare for their symptoms. Compared to the patients in the community and referral centres, patients at the tertiary hospitals and private clinics were younger, more likely to be working, and more often unmarried.

**Table 1:** Patient socio-demographic, healthcare seeking and pathologic characteristics

	Total N (%)	HSI 91 median (days)	DI 21 median (days)	CHC (*N* = 21) total (%)	RH (*N* = 26) total (%)	TH (*N* = 56) total (%)	PC (N = 21) total (%)
Age groups							
16–34	42 (33.9)	73	40	6 (28.6)	9 (34.6)	20 (35.7)	7 (33.3)
35–49	47 (37.9)	107	39	8 (38.1)	7 (26.9)	23 (41.1)	9 (42.9)
50–80	35 (28.2)	121	7	7 (33.3)	10 (38.5)	13 (23.2)	5 (23.8)
Occupation							
Housewife	55 (44.4)	156	11	15 (71.4)	14 (53.8)	21 (37.5)	5 (23.8)
Public Service	19 (15.3)	29	7	1 (4.8)	3 (11.5)	10 (17.9)	5 (23.8)
Business	9 (7.3)	107	8	0 (0)	2 (7.7)	5 (8.9)	2 (9.5)
Student	9 (7.3)	15	170	1 (4.8)	1 (3.8)	5 (8.9)	2 (9.5)
Other	32 (25.8)	67	138	4 (19.0)	6 (23.1)	15 (26.8)	7 (33.3)
Civil status							
Married	83 (66.9)	90	21	19 (90.5)	18 (69.2)	32 (57.1)	14 (66.7)
Single	14 (11.3)	70	49	0 (0)	1 (3.8)	10 (17.9)	3 (14.3)
Divorced	7 (5.6)	28	140	0 (0)	3 (11.5)	4 (7.1)	0 (0)
Widowed	20 (16.1)	245	7	2 (9.5)	4 (15.4)	10 (17.9)	4 (19.0)
Residence							
Bamako	49 (39.5)	107	32	8 (38.1)	11 (42.3)	16 (28.6)	14 (66.7)
Other	75 (60.5)	73	19	13 (61.9)	15 (57.7)	40 (71.4)	7 (33.3)
Health Insurance							
Yes	38 (30.6)	31	18	3 (14.3)	10 (38.5)	18 (32.1)	7 (33.3)
No	86 (69.4)	146	29	18 (85.7)	16 (61.5)	38 (67.9)	14 (66.7)
Knowledge about BSE							
Yes	37 (29.8)	19	24	5 (23.8)	7 (26.9)	22 (39.3)	3 (14.3)
No	87 (70.2)	153	18	16 (76.2)	19 (73.1)	34 (60.7)	18 (85.7)
First Symptom Interpretation							
Cancer	30 (24.2)	23	25	3 (14.3)	7 (26.9)	12 (21.4)	8 (38.1)
Infection	27 (21.8)	38	49	4 (19.0)	2 (7.7)	17 (30.4)	4 (19.0)
Other	67 (54.0)	200	16	14 (66.7)	17 (65.4)	27 (48.2)	9 (42.9)
Visit Traditional healer before							
Yes	30 (24.2)	270	48	11 (52.4)	5 (19.2)	8 (14.3)	6 (28.6)
No	94 (75.8)	63	18	10 (47.6)	21 (80.8)	48 (85.7)	15 (71.4)
Reason for healthcare visit							
Knowledge of BC symptoms	35 (28.2)	5	24	2 (9.5)	7 (26.9)	20 (35.7)	6 (28.6)
Persistence. aggravation	76 (61.3)	186	16	18 (85.7)	14 (53.8)	31 (55.4)	13 (61.9)
Recommendation	13 (10.5)	247	22	1 (4.8)	5 (19.2)	5 (8.9)	2 (9.5)
Pathological Diagnosis							
Cancer	64 (51.6)	146	28	13 (61.9)	17 (65.4)	21 (37.5)	13 (61.9)
Cancer in situ	1 (0.8)	5	185	0 (0)	0 (0)	0 (0)	1 (4.8)
Benign	37 (29.8)	89	19	6 (28.6)	7 (26.9)	21 (37.5)	3 (14.3)
Inflammation	19 (15.3)	28	21	2 (9.5)	2 (7.7)	11 (19.6)	4 (19.0)
Other	3 (2.4)	255	5	0 (0)	0 (0)	3 (5.4)	0 (0)
Tumor Size*							
T0	3 (2.4)	28	0	1 (4.8)	0 (0)	2 (3.7)	0 (0)
T1	8 (6.5)	213	9	1 (4.8)	3 (11.5)	3 (5.6)	1 (5.0)
T2	34 (27.4)	31	23	4 (19)	5 (19.2)	18 (33.3)	7 (35.0)
T3	69 (55.6)	153	22	11 (52.4)	17 (65.4)	30 (55.6)	11 (55.0)
T4	7 (5.6)	183	25	4 (19.0)	1 (3.8)	1 (1.9)	1 (5.0)
Lymph node involvement**							
N0	62 (52.1)	107	19	5 (23.8)	18 (69.2)	28 (51.9)	12 (60.0)
N1	54 (44.6)	87	28	15 (71.4)	7 (26.9)	25 (46.3)	7 (35.0)
N2	4 (3.3)	193	21	1 (4.8)	1 (3.8)	1 (1.9)	1 (5.0)

HSI = Health seeking interval; DI = Diagnostic interval; CHC = Community Healthcare Centre, RH = Referral Hospital; TH = Tertiary Hospital; PC = Private Clinic; BSE: Breast Self-Examination; *3 records missing, **4 records are missing

**Fig. 1:**
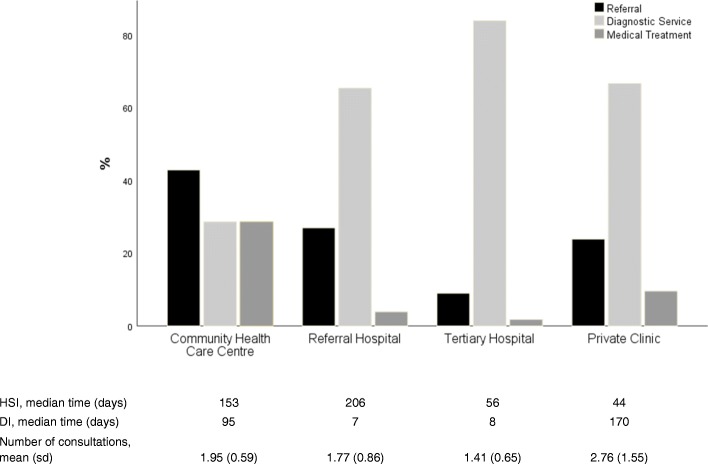
Health Seeking Interval (HSI), Diagnostic Interval (DI) and kind of recommendation according to first healthcare facility consultation

At the tertiary hospitals, a high proportion of patients resided outside Bamako, did not visit a traditional healer, and did not receive a breast cancer diagnosis. In contrast, at the private clinics, patients were typically from Bamako, visited traditional healers more often before visiting healthcare facilities or interpreting their symptoms as cancer. The HSI for patients visiting a community healthcare centre or referral hospital compared to patients initially seeking care at a tertiary hospital or private clinic was 3 to 4 times longer (Fig. 1).

The consecutive DI was shortest for patients of referral and tertiary hospitals, followed by patients of the community healthcare centres, and was longest for patients of private clinics. Women initially visiting community healthcare centres were referred in about 40% of all cases; only about 30% received a recommendation for mammography, ultrasound, or fine needle aspiration, compared to 60 to 80% of patients at the other facilities.

Results of the healthcare provider survey are presented in Table 2. The data show that medical doctors in the referral hospitals rated their knowledge about breast cancer better than did doctors based in community healthcare centres. However, in the community healthcare centres, doctors felt able to recognise suspected cases of cancer and typically referred patients directly to a referral or tertiary hospital.

**Table 2:** Personal information, breast cancer knowledge and referral practice in community healthcare centres and referral hospitals in Bamako

	All	CHC (N = 24)	RH (N = 12)
Age^1^ (mean, *SD*)	40.3 (6.0)	40.1 (6.9)	40.8 (4.1)
Years of work experience (mean, *SD*)	9.6	9.0	10.9
Gender			
female	7	7	0
male	29	17	12
Specialisation^1^			
Yes	14	2	12
No	21	21	0
Internship in oncology			
Yes	21	12	9
No	15	12	3
Number of suspected cases per month^2^			
0–1	27	20	7
2–6	4	3	1
Self-rated breast cancer knowledge			
Very good	10	2	8
Good	14	12	2
Middle	12	10	2
Poor	0	0	0
Frequency of performed CBE^1^			
Regularly	25	16	9
Rarely	9	7	2
Other	1	0	1
Able to recognise suspected cases			
Yes	33	21	12
No	1	1	0
Maybe	2	2	0
Recommendations for suspected cases^*^			
Further Analyses	22	10	12
Referral	17	17	0
Requested analyses for suspected cases*			
Fine needle aspiration/biopsy	16	8	8
Echography/Mammography	30	20	10
Other	1	0	1
Referral to which specialist if needed*			
Surgeon	13	6	7
Gynaecologist	21	14	7
Oncologist	19	14	5
Radiotherapy	1	0	1
No referral	1	1	0
Referral to Health care facility if needed*			
Referral Hospital	12	12	0
Private Clinic	0	0	0
Tertiary Hospital	26	15	11
No referral	1	0	1

^1^1 case missing, ^2^5 cases missing, *multiple answers possible; CHC = Community Health Care centre; RH = Referral Hospital; CBE = Clinical Breast Examination

**Fig. 2:**
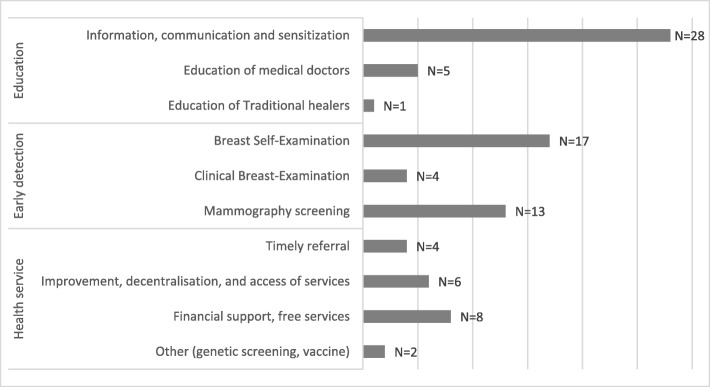
Improvement of early diagnosis and treatment as suggested by medical doctors in the community healthcare centres and referral hospitals in Bamako

Figure 2 depicts the frequency of suggestions for improving early detection or treatment of breast cancer and shows that the broad categories ‘education’ and ‘early detection’ were mentioned most commonly.

## Discussion

In Mali, patients are expected to enter the healthcare system at a low level, such as at the community healthcare centres and, if necessary, then be referred to a referral or tertiary hospital. One finding of our study is that this recommended patient pathway in the pyramidal healthcare system was not followed by the majority of patients receiving pathology services. Rather, almost half of all women entered the healthcare system directly at the third level, as they had chosen a tertiary hospital to initially seek out healthcare. This was especially true for women residing outside of Bamako, a fact that implies possible difficulties for patients with breast-related symptoms in these less populated regions to access the healthcare system at the community level. On average, only 57% of the population in Mali have access to healthcare within 5 km, with much lower proportions in some areas, compared to up to 93% in the capital, Bamako [21].

Any efforts to increase the demand for diagnostic services among women with breast-related symptoms nationwide could therefore lead to an excessive demand of the few specialised services at the tertiary hospitals in Bamako. The phenomenon of bypassing the community healthcare centres, and possible subsequent negative impacts on outpatient care at tertiary hospitals, was earlier described for other populations in sub-Saharan Africa [22, 23], indicating the lack of access, availability, and quality of primary healthcare services in many countries.

Our results also indicate that women of lower socio-economic status (e.g. no health insurance status, housewives) or with no breast cancer awareness first visited the community healthcare centres and had longer HSI. This suggests that the timely access to the healthcare system and the choice of the health care provider is determined by socio-economic factors and health literacy. Such inequalities might be exacerbated by awareness campaigns, in anticipation that women of higher socio-economic status might benefit most from same. Early detection programmes thus need to consider the structural barriers that exist in accessing optimal healthcare services for already deprived women and groups in the society. To deepen understanding of the women’s motivation to seek healthcare at a certain resource, it would be interesting to invest this in future mixed-method research studies.

The triage of women with any breast-related symptoms is a major challenge. The triple test score, combining results from clinical examination, mammography for postmenopausal women, and fine-needle aspiration biopsy, has been used to evaluate palpable breast masses and continues the recommended diagnostic approach, probably with additional use of ultrasound in special cases [24]. The Comprehensive Cancer Network Guidelines for basic resources [6] recommend patient history and clinical examination, followed by diagnostic mammography and fine needle aspiration, as a means of triaging women with breast-related symptoms. Fine needle aspiration is available only at the pathology service of 1 of the tertiary hospitals in Bamako, while imaging equipment is available at the referral hospitals and some private clinics. It is rarely available at the level of community healthcare centres in Mali. Therefore, the referral praxis of the community healthcare centres, shown in the results of our patient survey, reflects a well-working referral system for patients seeking healthcare initially at this level.

This result was supported by the results of our healthcare provider survey: the majority of physicians in the community healthcare centres in Bamako reported an ability to recognise possible breast cancer in patients; they routinely referred those women to hospitals. The longer diagnostic time intervals for women initially visiting community healthcare providers, compared to those presenting at referral or tertiary hospitals, might therefore partly be explained by a certain patient profile: older patients, without health insurance, who visited traditional healers before entering the healthcare system and who face further barriers (e.g. financial, waiting times and transport to another facility) in navigating through the healthcare system. For example, a study from Nigeria [25] estimated that out-of-pocket healthcare expenditures for non-communicable diseases are significantly higher in the lowest wealth quintile, compared to the three upper quintiles.

The role of the private clinics in providing access to health services and in triaging women with breast-related symptoms should be further investigated, since patients who initially visited a private clinic sought healthcare more quickly, but tended to need more time to receive a pathological diagnosis. A study from Uganda on referral of sick children at private facilities [26] highlighted several barriers at the provider and caretaker level, leading to prolonged time for treatment to begin. But generalising should be done with caution, since the term ‘private clinic’ encompasses a range of diverse clinics, from very small general services to highly specialised clinics. These should thus be differentiated in future studies.

### Limitations

Since this study only interviewed patients who received diagnostic service at the pathology department of a tertiary hospital, it excluded persons who did not seek healthcare or who discontinued their patient pathway after an initial healthcare visit – and thus never received a diagnosis for their breast-related symptoms. Patients outside Bamako might more often forgo medical care or further diagnostic services, as was found for health seeking for other non-communicable diseases in rural Nigeria [25]. Our study was generated as an explorative first step to developing appropriate hypotheses for future studies. Therefore, our findings need to be approached as preliminary only. They should be applied with caution in terms of generalising to other population groups and/or healthcare systems.

## Conclusions

In order to improve early diagnosis of breast cancer, it must be determined where women with breast-related symptoms should initially seek healthcare. If the community healthcare centres in Mali should remain the entry point for the majority of patients, services and access need to be strengthened – especially at the level of community healthcare centres outside of Bamako. Equipment such as ultrasound, as well as well-trained medical personnel, are needed to ensure appropriate triage and timely referral. However, barriers that keep women from seeking health services, especially at community healthcare centres (e.g. financial obstacles to obtaining diagnosis and also adequate treatment, waiting times, low quality of services, no trust in the medical system), also need to be addressed [11]. If, alternatively, referral hospitals and private clinics with gynaecological services serve as entry points for women with breast-related symptoms, these services need to be accessible and affordable for all women, independent of their socio-economic status and place of residence. In particular, relying on private healthcare providers to improve early breast cancer diagnosis – especially when they serve as entry points to the healthcare system – should be carefully monitored [27]. While early detection is important to improve survival rates of breast cancer patients, it should also be considered that barriers to accessing appropriate diagnosis and treatment, coupled with issues related to affordability of cancer care – both at the individual and national level – are still major bottlenecks to improving survival in most sub-Saharan African countries.

## Abbreviations

DI: Diagnostic Interval (Time between first healthcare visit and pathological diagnosis)
HIS: Health Seeking Interval (Time between first symptom recognition and first healthcare visit)
